# Computational approaches for direct cell reprogramming: from the bulk omics era to the single cell era

**DOI:** 10.1093/bfgp/elac008

**Published:** 2022-04-11

**Authors:** Andy Tran, Pengyi Yang, Jean Y H Yang, John Ormerod

**Affiliations:** School of Mathematics and Statistics, The University of Sydney, NSW, Australia; Charles Perkins Centre, The University of Sydney, NSW, Australia; School of Mathematics and Statistics, The University of Sydney, NSW, Australia; Charles Perkins Centre, The University of Sydney, NSW, Australia; Laboratory of Data Discovery for Health Limited (D24H), Science Park, Hong Kong SAR, China; School of Mathematics and Statistics, The University of Sydney, NSW, Australia; Charles Perkins Centre, The University of Sydney, NSW, Australia; Laboratory of Data Discovery for Health Limited (D24H), Science Park, Hong Kong SAR, China; School of Mathematics and Statistics, The University of Sydney, NSW, Australia

**Keywords:** cell reprogramming, gene regulatory network, single cell, modeling

## Abstract

Recent advances in direct cell reprogramming have made possible the conversion of one cell type to another cell type, offering a potential cell-based treatment to many major diseases. Despite much attention, substantial roadblocks remain including the inefficiency in the proportion of reprogrammed cells of current experiments, and the requirement of a significant amount of time and resources. To this end, several computational algorithms have been developed with the goal of guiding the hypotheses to be experimentally validated. These approaches can be broadly categorized into two main types: transcription factor identification methods which aim to identify candidate transcription factors for a desired cell conversion, and transcription factor perturbation methods which aim to simulate the effect of a transcription factor perturbation on a cell state. The transcription factor perturbation methods can be broken down into Boolean networks, dynamical systems and regression models. We summarize the contributions and limitations of each method and discuss the innovation that single cell technologies are bringing to these approaches and we provide a perspective on the future direction of this field.

## Introduction

Recent advances in direct cell reprogramming (also known as transdifferentiation) have transformed our perspective on cell development and regenerative medicine [1]. The ability to convert one cell type to another cell type has offered a potential treatment to many of the world’s major diseases. For example, type 1 diabetes is a result of the loss of insulin-producing beta cells in the pancreas, but recent studies have successfully reprogrammed these cells from pancreatic alpha cells, essentially curing type 1 diabetes in mice [2, 3].

Despite much attention to direct cell reprogramming, there have been substantial roadblocks hindering progress in this field [4]. Most importantly, current experiments are very inefficient, often producing <1% yield of the target cell type [5, 6]. Most protocols induce reprogramming by overexpressing a combination of transcription factors (TFs) [7, 8]. However, the optimal combination and levels of TF overexpression have historically been determined by trial and error, an expensive and time consuming approach [9, 10].

To this end, the field of direct cell reprogramming has greatly benefited from computational approaches that assist in the discovery of the combination of TFs and their overexpression levels for a desired cell conversion. Even to a small degree of accuracy, these predictions could guide the hypotheses to be experimentally validated, saving valuable time and resources.

Nevertheless, designing these computational models turns out to be a grand challenge in itself, as TFs can regulate each other in complex patterns. That is, finding the correct TF combination with optimal overexpression levels to drive the cell conversion depends on the complicated gene regulatory network (GRN), and hence it is a problem whose complexity grows exponentially. Thus, a variety of theoretical approaches have been developed to simplify this task by using different modeling techniques, leading to their own different advantages and disadvantages.

Until recently, most computational methods designed for modeling and prediction of cell reprogramming relied on ‘bulk’ expression profiles generated from a mixed population of cells or tissues. The advent of single-cell sequencing techniques [11, 12], however, is transforming the computational community, renewing our understanding of systems biology [13]. These methods have enabled us to observe the regulatory processes within cells at an unprecedented resolution, revealing the heterogeneity and stochasticity of cells [14, 15]. As such, it is expected that such data will significantly enhance our ability to develop more accurate computational methods which capture information that is unattainable from bulk data for direct cell reprogramming analysis.

**Figure 1:**
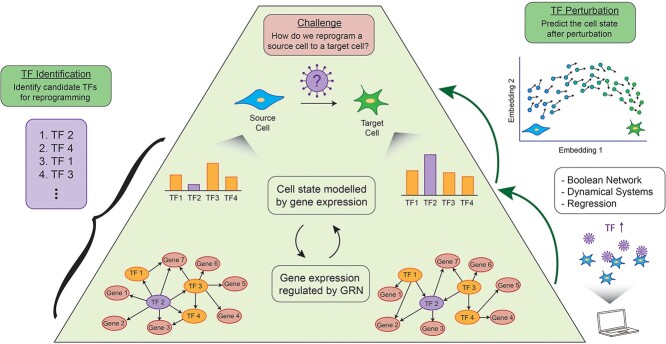
Overview of models for direct cell reprogramming. All methods aim to determine how to convert a source cell to a desired target cell. TF identification methods combine information from the gene expression and an estimate of the GRN to rank candidate TFs for reprogramming. TF perturbation methods (Boolean network, dynamical systems and regression) model the GRN to predict the change in gene expression resulting from a perturbation.

In this review, we categorize the current computational approaches for direct cell reprogramming into two main categories: TF identification [16–20], which aims to identify candidate TFs for a desired cell conversion, and TF perturbation, which aims to simulate the effect of a TF perturbation on a cell state (Figure 1). The TF perturbation methods can be broken down into Boolean networks [21–23], dynamical systems [24–26] and regression [27] models. We will not discuss the biological and technical challenges of direct cell reprogramming as they have already been covered in several excellent reviews [1, 4, 28, 29]. Instead, we assess the perspectives and limitations that each computational method offers with a discussion of how the rapid developments in single cell technologies are changing the way these methods model direct cell reprogramming. We hope that this review will guide the development of novel computational methods in this space, to exploit emerging data types and lead to new discoveries in direct cell reprogramming.

## Computational approaches based on bulk omics data

For the past few decades, microarray and RNA sequencing enabled researchers to profile the transcriptome for a bulk sample of cells. These technologies facilitated the development of a whole range of analyses, most notably differential gene expression [30], which identifies transcriptomic profiles associated with phenotypes of interest, leading to new insights in all fields of life science [31, 32]. In particular, these technologies led to the development of several models for direct cell reprogramming which we explore in this section.

### TF identification

This major category of approaches aims to identify candidate reprogramming TFs for a desired cell conversion. These approaches generally search for TFs which:

- are highly expressed in the target cell type;
- are lowly expressed in the source cell type;
- has target genes that are differentially expressed between target and source cell type.

The rationale here is that one would expect that candidate TFs should be overexpressed in the source cell type, and the downstream effect would bring the gene expression closer to that of the target cell type. This can be thought of as a differential gene expression analysis for the TFs which incorporate its target genes. This sort of analysis produces a score for each TF, which can be used to rank TFs by how likely they are to drive reprogramming. The existing TF identification methods, with their GRN estimation step and ranking algorithm, are summarized in Table 1.

**Table 1:** A comparison of TF identification methods for cell reprogramming.

Method	Year	Data	GRN Estimation	Ranking Algorithm	Applicability
D’Alessio *et al*.	2015	Gene expression (Microarray)	N/A	Jensen–Shannon Divergence to identify TFs that are highly expressed in a cell-type specific way.	Candidate TFs calculated for 233 tissue and cell types.
CellNet (Cahan *et al*.)	2014	Gene expression (Microarray)	Mutual information to build main network, community detection to identify subnetworks	GRN activity estimated for each subnetwork. Network Influence Score calculated to estimate impact on GRN activity.	Web application http://cellnet.hms.harvard.edu
Mogrify (Rackham *et al*.)	2016	Gene expression (FANTOM5 database: Microarray), protein–DNA interaction database (STRING and MARA)	Known interactions from protein–DNA interaction databases	Network Influence Score calculated to estimate importance for TF to regulate required genes.	Web application http://www.mogrify.net/
Lisa (Qin *et al*.)	2020	ChIP-seq, DNase-seq	Regulatory potential estimated from ChIP-seq	Identifying key regulators of differentially expressed genes based on regulatory potential	Web application http://lisa.cistrome.org
ANANSE (Xu *et al*.)	2021	Gene expression (RNA-seq), chromatin accessibility (ATAC-seq), ChIP-seq	Regulatory potential estimated from ChIP-seq	Network Influence Score calculated to estimate importance for TF to regulate required genes.	Key TFs identified for 18 tissues Python package available on GitHub

These methods are greatly advantageous as it significantly reduces the TFs that need to be experimentally validated. Indeed, TF identification methods have successfully recapitulated many known TFs to drive direct cell reprogramming [16–20] and they have guided the discovery of several novel cell conversions such as fibroblasts to retinal pigment epithelial cells [17] and fibroblasts to keratinocytes [18]. Here, the approaches can be broadly grouped into whether they estimate the GRN, and if so, whether they incorporate other sources of information, such as protein–DNA interaction or ChIP-seq data.

The earliest approaches only compared TF expression to infer the candidate TFs for cell reprogramming. An early example is the work by D’Alessio and colleagues [17] where they search for candidate TFs that are not only highly expressed in the target cell, but also in a cell-type specific fashion, using a Jensen–Shannon Divergence metric to rank the TFs. However, this approach only captures overall differences in the expression of each TF and does not consider their regulatory effect. This can be an issue for direct cell reprogramming where it is the downstream effects of TF overexpression that determines successful reprogramming.

Instead, one should aim to identify candidate TFs by incorporating the GRN structure. An early work in this category is CellNet [16], which estimated the GRN from gene expression data only. Here, the authors use a mutual information-based algorithm on the gene expression data to estimate the target genes for each TF, forming the basis of the GRN. This allows a Network Influence Score to be calculated which ranks the importance of each TF in perturbing the GRN to match the desired target state. However, only using gene expression data as the main input poses another challenge, due to the highly correlated nature of gene expression. In other words, TFs will be associated with any highly correlated genes, which may not be representative of reality where there may be another driver TF that is regulating them both. This is a critical component for direct cell reprogramming, as the grand challenge is to identify the key TFs which drive the downstream effects to result in successful reprogramming.

To tackle this issue, more recent techniques incorporate information from other sources to better estimate the GRN. For example, Mogrify [18] takes a similar approach to CellNet, but uses external protein–DNA interaction databases to estimate the GRN. This way, the TF-gene regulations that are considered are only those which have been experimentally validated or are predicted to exist. Lisa [19], unlike the previous methods that used gene expression data, uses ChIP-seq data which measures the binding affinity of each TF to *cis*-regulatory elements. For a given gene, the binding affinities from nearby *cis*-regulatory elements can be aggregated to calculate a regulation potential for each TF to each gene. Candidate TFs are then ranked by their potential to regulate the differentially expressed genes between the source and target cell types. ANANSE [20] combines ideas from both Mogrify and Lisa, building a base GRN from both gene expression and ChIP-seq data. TFs can then be ranked by a Network Influence Score, measuring how likely they are to regulate key genes for cell reprogramming.

The key output from all of these approaches is a ranked list of candidate TFs for a desired cell conversion. Although useful, TFs rarely work alone and so there would still be many quantities and combinations of TFs left to be experimentally validated. This is further hindered by false positives that may appear in the ranked list, due to the correlated nature of gene expression and the vast number of TFs and genes. Another significant limitation is that TF identification methods only consider the initial and final cell states and so do not consider the intermediate cell states or the trajectory during cell reprogramming. This motivates the need for a computational model for direct cell reprogramming that can estimate the GRN dynamics and predict the effect of perturbing TF concentrations.

### TF perturbation

Indeed, the second major category of approaches aims to complement cell reprogramming research by computationally predicting the effect of overexpressing a TF onto the cell state. This can then be used to test TF combinations, finding key combinations and quantities for a desired cell conversion. This type of modeling has the added advantage of being able to analyze the gene and cell state transition trajectory and cell state attractors during cell conversion. Considering the regulatory role of TFs, these methods begin by producing some model to estimate the GRN structure, which is then used to predict the effect of TF overexpression. With the numerous mathematical approaches to GRN inference [33], there are a few subcategories of TF perturbation methods which are based on different theoretical models. These models offer the advantage of making predictions with varying assumptions and biological interpretations. The existing TF perturbation methods are summarized in Table 2.

**Table 2:** A comparison of TF perturbation methods for cell reprogramming.

Method	Year	Category	Data	GRN Estimation	Applicability
Hopfield Neural Networks (Lang *et al*.)	2014	Boolean network	Gene expression (Microarray), Domain knowledge	Waddington’s epigenetic landscape estimated from experimental data.	Theoretical Model
Toggle Switches (Okawa *et al*.)	2016	Boolean network	Gene expression (Microarray)	Normalized ratio difference of TF pairs to identify toggle switches.	Theoretical Model
IQCELL (Heydari *et al*.)	2022	Boolean network	scRNA-seq	Mutual information of gene expression from cells sorted by pseudotime.	Python package available on GitLab
Del Vecchio *et al*.	2017	Dynamical systems (ODE)	Domain knowledge	ODE parameters estimated from experimental literature.	Theoretical Model (simulation)
Ronquist *et al*.	2017	Dynamical systems (difference equation)	Time series RNA-seq	Transition matrix estimated from time series data. Regulatory effect estimated by the number of TFBSs.	Theoretical Model Patented
Rommelfanger *et al*.	2021	Dynamical systems (ODE)	Domain knowledge	ODE parameters estimated from experimental literature.	Theoretical Model (simulation) Simulation code in Julia available on GitHub
CellOracle (Kamimoto *et al*.)	2020	Regression	scRNA-seq, scATAC-seq	All possible regulations filtered from scATAC-seq and TF motif data, then further filtered from regression with scRNA-seq data.	Python package available on GitHub

### Modeling with Boolean networks

Some of the first approaches to model GRN dynamics came in the form of Boolean networks, where the activity of each gene is simplified into an ‘on’ or ‘off’ state [34]. This way, every gene is represented as a node in a network, and regulatory relationships between TFs and genes are represented as a directed edge. The network then follows fixed rules which govern how each state of the network leads to a future state, often with a biological interpretation. For example, if the *GATA1* gene is switched ‘on’, this could trigger the *PU.1* gene to be switched ‘off’. As there are only finitely many possible states, one can find steady states or cycles which reflect stable cell states, and the downstream effect of perturbing TFs on these states can then be modeled. These types of approaches have successfully contributed to the modeling of a number of reprogramming and differentiation processes like neuronal differentiation [22], reprogramming to pluripotency [21] and T-cell and red blood cell development [23].

The challenge of fitting a Boolean network model is to determine the dynamics between possible states. Lang and colleagues [21] achieve this by assigning each state an ‘energy’ by using known results from experimental literature, natural cell cycle dynamics and external conditions. Cell states can then be perturbed and are then expected to transition from high energy states to low energy states, following Waddington’s landscape model for cell differentiation [35]. However, relying on published results may lead to bias in the model as undiscovered interactions cannot be used. Okawa and colleagues [22] overcome this by inferring the dynamics using the theory of toggle switches where in a progenitor cell, an antagonistic TF-pair pushes the cell state into one of two cascades, leading to different cell lineages. This is done by comparing the ratio of expression of TF-pairs between progenitor and daughter cell types to identify potential toggle-switches. However, this means that this method is only able to model these lineage specifiers, and not the entire GRN dynamics.

In summary, Boolean network models offer a simple framework to understand and model the regulatory relationships between genes. However, the Boolean assumption of each gene being in either an on or off state is a crude oversimplification of real regulatory dynamics. Some cell fate decisions may require genes to be expressed at specific intermediary levels which cannot be modeled in a Boolean network [22]. Furthermore, the number of possible states of the network grows exponentially with the number of genes included, making it difficult to model more complex scenarios with many genes.

### A dynamical systems approach to reprogramming

A different and more detailed model for the cell reprogramming process would be a dynamical systems approach, which aims to model the chemical and physical properties that control gene expression. This will generally include upregulation or downregulation of a gene from any regulatory TFs, and a degradation rate in which the mRNA in a cell is constantly decaying. By writing all of these components into an equation, we are able to model how TF expression is varying over time with a biologically meaningful interpretation for the model. These equations can be solved, either numerically through simulations, or analytically using dynamical systems theory. This reveals the steady states of the dynamical system, which corresponds to stable cell states and their stability.

The time can be treated as continuous, which can be modeled with ordinary differential equations (ODEs). For example, Del Vecchio and colleagues [24] devise a blueprint for a genetic feedback controller and model the cell reprogramming process using ODEs. Here, the rate of change in gene expression $\frac{d{x}_i}{dt}$ is written in terms of the degradation rate $\gamma$, an external TF input $u$ and the Hill function $H$, which captures the regulation of TF $x$ by other TFs.

$$ \frac{d{x}_i}{dt}={H}_i\left(\boldsymbol{x}\right)-{\gamma}_i{x}_i+{u}_i. $$

They use this to model the dynamics of a two-TF system in reprogramming to pluripotency, revealing three steady states: the trophectoderm, primitive endoderm and pluripotency. Under this ODE model, they theoretically show that some TFs may need to be expressed at an intermediary level for a desired cell conversion. This demonstrates the limitations of Boolean Network models which assume that genes can only be in an on or off state.

Alternatively, time can be considered in discrete steps, which can be modeled using difference equations. For example, Ronquist and colleagues [25] use difference equations to incorporate the natural cell cycle dynamics when modeling the effect from external perturbations. Here, ${x}_{k+1}$(the cell state at time $k+1$) is written in terms of ${x}_k$ (the cell state at time $k$), ${A}_k$ (the transition matrix), ${u}_k$ (the external TF input) and $B$ (the effect that each TF has on the gene expression). Here, $B$ is estimated by the number of TF binding sites on topologically associating domains, identified with Hi-C data.

$$ {x}_{k+1}={A}_k{x}_k+B{u}_k $$

They employ this model to identify the optimal timing and combination of TFs for a desired cell conversion. They predict that some TF combinations have a preference for being introduced at the start of the cell cycle, whereas others have a preference toward the end. This highlights the importance of considering the timing when performing direct cell reprogramming as the cell state constantly changes over time.

These dynamical systems models provide an excellent framework to study how TF expression evolves over time. Furthermore, their parameters can be closely linked to biological mechanisms, leading to interpretable results. The main limitation is the challenge associated with estimating the parameters of large dynamical systems models. This is because these parameters are often determined from results in experimental literature, or they need to be estimated with time course data which may not be readily available in public databases. Even if one were to generate their own data, this would take additional time and resources, and narrows the options for source cell types to those used in the experiment.

## Computational approaches in the single cell era

A significant limitation of all the computational methods based on bulk omics data is that they assume that the initial cell population is homogeneous, so that the cells will all respond to perturbations in an identical way. This limitation in approaches exists as a consequence of the available technology at the time, where bulk sequencing data only provide a general average of the cell population.

However, the advent of single cell technologies has provided an unprecedented resolution into the biological mechanisms within individual cells. In particular, these technologies reveal the heterogeneity and stochasticity of cells [14, 15], which can explain the significant inefficacy of current direct cell reprogramming experiments, often resulting in several distinct clusters of unsuccessful conversions [15]. Thus, by modeling direct cell reprogramming at the single cell level, we would be able to develop a model that is more representative of reality, better identifying the TFs for a desired cell conversion.

Given the recency of single cell technologies, there are currently few methods in this space, but we expect the number to significantly increase in the coming years.

### Novel techniques for TF identification with single cell

TF identification methods can benefit from single cell data as it gives us access to the distribution of gene expression across cells. Although we are yet to see any novel methods in this area, there are certainly potential extensions of existing algorithms. As these methods were mostly based on differential expression methods, single cell data provide the opportunity to use ideas from more recent single cell differential expression methods like MAST [36] which uses a generalized linear model to address the specific biases seen in single cell data. These data also opens up the opportunity for other analyses that depend on the gene expression distribution like differential variability [37], differential distribution [38] and differential stability [39], which may identify TFs with different properties that could be novel candidate TFs for direct cell reprogramming.

Furthermore, single cell data have allowed the development of more advanced GRN inference tools, like SCENIC [40] which uses a random forest regression to identify coexpressed TF-gene pairs, which are then trimmed motif enrichment. By doing this, SCENIC can build regulons for each TF, which identifies complex regulatory patterns that are biologically possible. This could be a useful extension to TF identification methods which calculate a network influence score, such as CellNet, Mogrify and ANANSE, where a more refined estimate could lead to a more accurate and interpretable model.

### Boolean networks made scalable with single cell data

**Figure 2:**
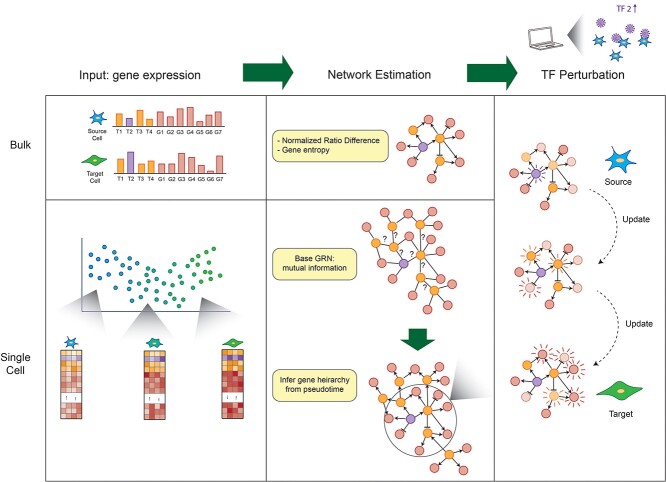
Single cell data allow cells to be sorted in pseudotime, enabling gene hierarchies to be estimated at a larger scale than bulk data. However, the Boolean assumption remains the same.

Another innovation that single cell data bring is the ability to observe cells along the continuum of a biological process like cell cycle, cell differentiation or cell reprogramming. A variety of trajectory inference methods [41, 42] have been developed to infer a pseudotime, constructing an order of the cells along these dynamic processes. Recently, IQCELL [23] exploits this application of single cell data to bypass the scalability issue of Boolean Networks, estimating the regulatory relationships in a developmental system in an unbiased way. Here, they use mutual information on the scRNA-seq data to establish the interaction network, and a gene hierarchy is built using pseudotime to infer the order of gene regulation (Figure 2). This innovative approach allows Boolean Networks to be extended to much larger systems and recapitulated several known results about perturbations on T-cell development. However, the oversimplifying assumption of each gene in an on or off state will always remain a limitation of Boolean networks.

### Dynamical systems capture heterogeneity at the single cell resolution

**Figure 3:**
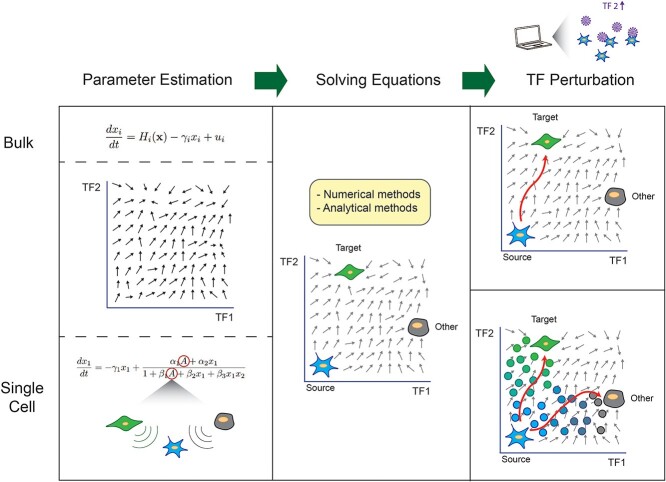
Single cell data allow dynamical systems models to account for heterogeneity in the cell population, more accurately resembling experimental results. However, estimating the parameters remains a challenge.

To account for the heterogeneity in direct cell reprogramming populations as revealed by single cell data, Rommelfanger and colleagues [26] generalize the ODE framework to create a model at the single cell level. They achieve this by incorporating additional terms into the ODEs which represent how cells send a ‘be like me’ signal to other cells which helps to coordinate cell fate decisions during differentiation (Figure 3). In particular, this interpretable model captures the biological mechanisms behind the bifurcation in lineage commitment.

They use the following ODEs (with notation adapted):

$$ \frac{d{x}_1}{dt}=-{\gamma}_1{x}_1+\frac{\alpha_1A+{\alpha}_2{x}_1}{1+{\beta}_1A+{\beta}_2{x}_1+{\beta}_3{x}_1{x}_2} $$

$$ \frac{d{x}_2}{dt}=-{\gamma}_2{x}_2+\frac{\alpha_3B+{\alpha}_4{x}_2}{1+{\beta}_4B+{\beta}_5{x}_2+{\beta}_6{x}_1{x}_2+{\beta}_7{x}_1{x}_3} $$

$$ \frac{d{x}_3}{dt}=-{\gamma}_3{x}_3+\frac{\alpha_5{x}_1}{1+{\beta}_8{x}_1+{\beta}_9C} $$

where changes in TF concentrations ${x}_1$, ${x}_2$ and ${x}_3$, are written in terms of the degradation rates ${\gamma}_i$, the activation rates ${\alpha}_i$, inhibition rates ${\beta}_i$ and external signals $A$, $B$ and $C$, which arise from cell–cell communication.

They successfully apply this to model the antagonistic TF pair *GATA1* and *PU.1* in the commitment of a myeloid progenitor cell to the erythroid/megakaryocyte lineage or granulocyte/monocyte lineage. This application demonstrates the versatility of dynamical systems to model physical processes with meaningful interpretations. However, the reliance on estimating parameters with prior knowledge continues to be a limitation to extending dynamical systems models to larger systems, as in this case, the model with three TF concentrations required 17 parameters to be estimated.

### Regression models added to the toolkit in the single cell era

The curation of large single cell data sets opens up a new category of algorithms, regression, which can infer complex patterns in data. A regression algorithm uses a training data set to build a model for a response variable, given several explanatory variables. In our case, we would be interested in modeling the gene expression of a cell using the TF expression. Although this approach could be applied to samples of bulk RNA-seq data, it is able to better exploit the extra resolution and scale offered by single cell data, inferring the patterns between the TF expression and gene expression. For example, CellOracle [27] uses scATAC-seq data to first build a base GRN of possible TF-gene regulations, and then fits a linear model for the gene expression $y$ using the list of remaining TFs $X$.

$$ {\boldsymbol{y}}_j=\alpha \mathbf{1}+\beta \boldsymbol{X}+\varepsilon . $$

The fitted coefficients of this model, $\beta$, can be used to predict the effect of perturbing a TF, that is a change in $X$ (TF expression) will have a corresponding change in $y$ (gene expression). Furthermore, this can be applied to each single cell in the data, accounting for the heterogeneity of the initial cell population. Considering all the predicted changes in cell state, the result of TF perturbation can be interpreted as the dynamics of RNA which describes the general shift in gene expression.

Regularization like LASSO and Ridge Regression can be introduced to reduce the number of important predictors in the final model, as in CellOracle [27]. The vast statistical theory behind regression analyses allows many variations on regression-based methods [43]. Linear models have the advantage of being efficient and interpretable, but more advanced models can capture complex nonlinear relationships. These regression approaches can also scale up easily with more genes or cells.

However, regression models can be vulnerable to bias in scRNA-seq data. In the sequencing protocol for scRNA-seq, lowly expressed genes may not be captured in the amplification step causing them to be completely unrepresented in the final data [44, 45]. This means that a regression approach would be unable to model these TFs, and dropouts which are missing not at random would lead to bias in the fitted coefficients.

### Novel data modalities offer a deeper insight into direct cell reprogramming

The methods discussed thus far have focused heavily on gene expression to develop predictive models. However, a challenge in the modeling of direct cell reprogramming is that it depends heavily on the cell’s gene regulation which is influenced by many other factors such as DNA accessibility [46], micro-RNAs [47] and small molecules [48]. In recent years, the bioinformatics community has witnessed the development of novel types of sequencing data and the expansion of databases which hold the potential to more accurately infer the biological processes behind direct cell reprogramming. For example, the continuously expanding LINCS database [49] catalogs the transcriptional responses of multiple cell lines to a wide variety of drugs. Napolitano and colleagues used this to develop DECCODE [50], a computational method to identify drugs that may increase the efficacy of direct cell reprogramming experiments by incorporating their transcriptional responses. They validate this on reprogramming fibroblasts to human-induced pluripotent stem cells, and curate a list of predicted drugs that may facilitate a range of cell conversions.

Other data types have a large potential to be incorporated into computational direct cell reprogramming models. Perturb-seq [51] is a method that can scalably measure the effect of gene knockouts on overall gene expression at the single cell level, which could help to infer the effect of TF perturbations. Single cell multiomics sequencing technologies [52] offer greater insight into the intracellular dynamics of single cells by simultaneously sequencing different modes of the gene regulatory process. For example, a few recent techniques can capture RNA expression and chromatin accessibility within the same cell [53, 54] and integrating these different modalities of gene regulation will likely provide a more accurate model for direct cell reprogramming. Furthermore, single cell spatial transcriptomics like Visium [55] or MERFISH [56] hold the potential to incorporate information about the local microenvironment, where the position of cells in 3D space has been implicated to play a key role in cell differentiation [57]. This could be a major cause for the heterogeneous outcomes seen in cell reprogramming experiments and may provide guidance on how to improve the efficacy of cell reprogramming.

## Conclusions and perspectives

Building a computational model for direct cell reprogramming is a difficult yet valuable task. In this review, we assessed how TF identification and TF perturbation methods have sought to take on this challenge and how single cell technologies have led to the innovative applications of these approaches. We see that no one type of approach is the best model for direct cell reprogramming, but rather they each bring a different perspective to the biological processes of cell reprogramming, with their own advantages and limitations.

TF identification methods create a list of candidate TFs for reprogramming, with single cell data offering more potential techniques, but they are unable to predict the result of a cell reprogramming experiment. TF perturbation methods aim to model the effect of a TF perturbation and can be broken down into three subcategories. Boolean networks effectively model simple gene regulatory dynamics, and single cell data allow the unbiased estimation of these dynamics. However, these may not generalize to complex situations due to the simplification of gene states into an on or off state. Dynamical systems provide a rigorous and interpretable framework to model TF expression over time and can be extended to model cell populations at the single cell level. However, they can be difficult to fit for larger models as their parameters are often chosen from experimental literature. Regression models are able to infer trends and make predictions at the single cell level, accounting for the diversity of cell populations, but are vulnerable to biases in single cell data.

As single cell sequencing technologies continue to improve, becoming more accessible and capturing more modalities, we expect this trend to drive a new wave of more sophisticated computational approaches for direct cell reprogramming, taking on all different categories of approaches. Considering the pace at which both the technology and models are being developed and improved, we foresee that future methods will continue to accelerate our understanding of direct cell reprogramming and its translation into a clinical therapy.

**Key Points**

- Computational approaches for cell reprogramming can be grouped into TF identification methods and TF perturbation methods (Boolean networks, dynamical systems and regression).
- Single cell data enable significant innovations in all categories.
- Single cell multiomics data are likely to lead a new revolution in computational approaches for cell reprogramming.

## Author Biographies

**Andy Tran** is a PhD Student at the School of Mathematics and Statistics, the University of Sydney. He is a member of the Sydney Precision Bioinformatics Research Alliance.

**Pengyi Yang** is a Senior Lecturer at the School of Mathematics and Statistics, the University of Sydney. He is a member of the Sydney Precision Bioinformatics Research Alliance.

**Jean Yang** is a Professor at the School of Mathematics and Statistics, the University of Sydney. She is a member of the Sydney Precision Bioinformatics Research Alliance.

**John Ormerod** is an Associate professor at the School of Mathematics and Statistics, the University of Sydney. He is a member of the Sydney Precision Bioinformatics Research Alliance.

## Notes

The authors are members of the Sydney Precision Bioinformatics Research Alliance that develops bioinformatics methods.
